# Economies and Sources of Income

**Published:** 1921-09-17

**Authors:** 


### THE LONDON HOSPITAL.

### Economies and Sources of Income.

The quarterly report of the House Committee of the London Hospital is a record of activity in many directions. The chief points from the report are given below.

### Recent Economies.

The wards reported closed at the last Court are still lying empty, and no hope can be given of their reopening this year. Indeed, although the financial position is less strained than it was three months ago, it is impossible to fix any date at which the London Hospital will be able to take up its full load of work. To do so either the expenditure must be reduced or the income of the hospital must be increased.

To the reduction of expenditure the Committee has given most serious attention. It is considering, for instance, the substitution of oil fuel for coal in the main furnaces of the hospital, and is obtaining expert advice on the subject.

Telephone lines have been cut down by about half, and an automatic system has been introduced for speaking from ward to ward without the calls passing through a central exchange. This has enabled the hospital to dispense with the large telephone staff supplied by the Post Office, and in their place the Committee has engaged two blind soldiers from St. Dunstan's, who are most efficiently carrying out their duties. To save expense night operators have been dispensed with. With lines reduced from ten to six, with "talking-out" instruments reduced from forty-five to twenty-three, with a twenty-four-hour staff reduced to a twelve-hour staff, it is impossible to give as prompt service as of old, and the public is asked to be patient.

Close attention has been given to the reduction of the cost of drugs and of dressings—a very important part of hospital expenditure. The drugs which may be given to out-patients have been restricted, and a great many expensive remedies may now only be ordered on the signature of a member of the visiting staff; the stock of medicines allowed in the wards has been cut down, and none but the barest necessaries are permitted; the staff is considering whether less costly substitutes can be found for some of the expensive materials used in surgical operations.

### A Limited Economy.

The closing of the beds has brought some economies, but "nothing like as much as would, at first sight, be expected." Two hundred beds represent about a quarter of the total occupied beds, but the reduction cannot thereby reduce the cost of the hospital by a quarter. Cost of food, drugs, laundry, and cleaning is, of course, lower, but the standing charges of a hospital are very heavy, and they are not affected unless the work is very drastically and permanently reduced.

### Shorter Hours for Night Nurses.

The report draws attention to the fact that the closing of these beds, and the consequent distribution of the nurses throughout the hospital, has enabled the Matron to shorten the hours of the night nurses. They now go off duty at 7.20 in the morning instead of 9.20, and so get a long morning off duty before going to bed at 1. This is an improvement which the Committee had long been trying to bring about.

### Charity and Revenue.

The increase of income is more hopeful. "To those who think that the age of charity is over we should like to point out that, although the past three months is usually considered the most difficult period in the year to raise funds for charitable objects, never has the hospital appealed more to the generous than during this difficult time." The bazaar given entirely by the nursing staff was an extraordinary success. It was organised by a committee of sisters and nurses and probationers. The result of that one day, the end of many days of hard work, was that a sum of £7,000 has already been handed to the hospital, and more has yet to come. Messrs. Lyons generously supplied the whole of the refreshments free of all cost, and also the staff to serve them. Articles left over from the bazaar have been purchased by the nurses themselves. The Prince of Wales sent a replica of the Cenotaph at Whitehall, which the nurses bought and presented to the hospital. It will be set up at the entrance hall with the list of the London Hospital staff and students who gave their lives in the war—about 140.

The East End Hospitals Demonstration, when processions toured the streets in the East End, brought a sum of £800 to the hospital. The report also contains an impressive list of undertakings to help the hospital not only in London but also in the provinces. The Committee draws attention to these to show how much is being done by charitable people on behalf of our hospital.

### A Year of Patients' Payments.

Nevertheless, the constant increase in the cost of medical and surgical work indicates to the Committee that something more is required than voluntary gifts. In ten years the cost per bed has increased from £1 19s. 2d. per week to £4 18s. 9d. Since the decisions, in June of last year, to ask all in-patients to contribute a sum of one guinea a week towards the cost of their maintenance, the sum of £20,000 was paid in the year ending June 28, 1921.

In regard to the decision of St. Thomas's, the Royal Free, and the London Hospital to try for a year an adaptation of the provident scheme known as the Sussex Scheme, which provides its members, in consideration of annual premiums, with advantages which include a home nursing service and an ambulance service, a start will be made towards the end of the year. Meanwhile the arrangements are being made by a committee, consisting of the Hon. Sir Arthur Stanley, Lord Dawson (Chairman of the Consultative Council of the Ministry of Health, and a member of the London Hospital staff), Sir Alan Anderson, a member of the Policy Committee of the King's Fund, Sir Napier Burnett, Mr. McAdam Eccles, surgeon at St. Bartholomew's Hospital, and Dr. Gordon Dill, senior physician of the Sussex General Hospital.

